# Treatment of Periodontal Ligament Stem Cells with MOR and CBD Promotes Cell Survival and Neuronal Differentiation via the PI3K/Akt/mTOR Pathway

**DOI:** 10.3390/ijms19082341

**Published:** 2018-08-09

**Authors:** Veronica Lanza Cariccio, Domenico Scionti, Antonio Raffa, Renato Iori, Federica Pollastro, Francesca Diomede, Placido Bramanti, Oriana Trubiani, Emanuela Mazzon

**Affiliations:** 1 IRCCS Centro Neurolesi “Bonino-Pulejo”, Via Provinciale Palermo, Contrada Casazza, 98124 Messina, Italy; veronica.lanzacariccio@irccsme.it (V.L.C.); domenico.scionti@gmail.com (D.S.); antoniraffa@hotmail.it (A.R.); placido.bramanti@irccsme.it (P.B.); 2 Consiglio per la Ricerca in Agricoltura e L’analisi Dell’economia Agraria, Centro di Ricerca Agricoltura e Ambiente (CREA-AA), Via di Corticella 133, 40128 Bologna, Italy; renato.iori48@gmail.com; 3 Dipartimento di Scienze del Farmaco, Università del Piemonte Orientale, Largo Donegani 2, 28100 Novara, Italy; federica.pollastro@uniupo.it; 4 Department of Medical, Oral and Biotechnological Sciences, University “G. d’Annunzio” Chieti-Pescara, 66100 Chieti, Italy; francesca.diomede@unich.it (F.D.); trubiani@unich.it (O.T.)

**Keywords:** periodontal ligament mesenchymal stem cells (hPDLSCs), moringin (MOR), cannabidiol (CBD), apoptosis, PI3K/Akt/mTOR pathway, Next Generation Sequencing (NGS), transcriptome analysis

## Abstract

Periodontal ligament mesenchymal stem cells (hPDLSCs), as well as all mesenchymal stem cells, show self-renewal, clonogenicity, and multi-tissue differentiation proprieties and can represent a valid support for regenerative medicine. We treated hPDLSCs with a combination of Moringin (MOR) and Cannabidiol (CBD), in order to understand if treatment could improve their survival and their in vitro differentiation capacity. Stem cells survival is fundamental to achieve a successful therapy outcome in the re-implanted tissue of patients. Through NGS transcriptome analysis, we found that combined treatment increased hPDLSCs survival, by inhibition of apoptosis as demonstrated by enhanced expression of anti-apoptotic genes and reduction of pro-apoptotic ones. Moreover, we investigated the possible involvement of PI3K/Akt/mTOR pathway, emphasizing a differential gene expression between treated and untreated cells. Furthermore, hPDLSCs were cultured for 48 h in the presence or absence of CBD and MOR and, after confirming the cellular viability through MTT (3-(4,5-dimethylthiazolyl-2)-2,5-diphenyltetrazoliumbromide) assay, we examined the presence of neuronal markers, through immunofluorescence analysis. We found an increased expression of Nestin and GAP43 (growth associated protein 43) in treated cells. In conclusion, hPDLSCs treated with Moringin and Cannabidiol showed an improved survival capacity and neuronal differentiation potential.

## 1. Introduction

Human adult mesenchymal stem cells (MSCs) retain the characteristic of all stem cells to generate different mature cell lines and self-renewal propriety. These cells can be obtained from different tissue sources such as bone marrow, adipose tissue, and peripheral blood [1]. Another source of adult stem cells, which involve less invasive extraction techniques, is represented by oral and dental tissues, such as apical papilla [2], gingiva, dental pulp [3], exfoliated deciduous teeth [4], dental follicle, and periodontal ligament (hPDLSCs) [5,6,7,8]. Mesenchymal stem cells derived from the oral cavity are able to differentiate in vitro, also at long-term passage, resulting easy to manipulate and to use in laboratory practice [9]. Self-renewal capacity, differentiation, and immunomodulatory proprieties of human MSCs provide new avenues for cell-based therapy in regenerative medicine [10,11,12,13].

A pretreatment of stem cells with substances that show antioxidant, anti-inflammatory and anticancer proprieties can improve their survival in the re-implanted tissue of patients and can influence a preferential differentiation into a specific cell lineage. Our research group has extensively investigated the effects of two phytocompounds, Moringin (4-(α-l-rhamnosyloxy)-benzyl isothiocyanate-MOR) and Cannabidiol (CBD), on stem cell culture. MOR is the principal active constituent obtained from the extract of *Moringa oleifera* (fam. *Moringaceae*) seeds. We have conducted numerous studies which demonstrated anti-inflammatory proprieties of this compound both in vivo [14,15] and in vitro experiments [16], and recently, we have demonstrated its capacity to improve differentiation of periodontal ligament stem cells to osteocytes, chondrocytes, adipocytes, and neuronal cells [17]. Also CBD, the active phytocannabinoid extract from *Cannabis sativa*, was largely studied for its anti-inflammatory and antioxidant proprieties. Our research in anti-inflammatory activity of CBD was conducted both in macrophage culture [18] and in human gingiva-derived mesenchymal stem cells (hGMSCs) [19]. The effects of these two compounds have always been investigated when they are used alone and not in combination. Our only research in which the effects of combined treatment had been investigated, had been performed on macrophages rather than on stem cells. In this previous research, we had seen the anti-apoptotic effect of combined use of MOR and CBD on LPS-stimulated macrophages, by immunohistochemical analysis for detection of Bax and Bcl-2 expression, noting a decreased level of Bax, Bcl-2, and caspase-3 [18].

In the present study, we have investigated more accurately the anti-apoptotic or proliferative effect of combined treatment with MOR and CBD on hPDLSCs, analyzing their transcriptomic profile by Next Generation Sequencing analysis (NGS). In particular, we investigated the possible involvement of PI3K/Akt/mTOR pathway in stem cell survival, by enhanced expression of anti-apoptotic genes and contemporary inhibition of pro-apoptotic genes. Moreover, a longer survival time in the transplanted tissue becomes important both for the success of the cell-therapy and for the increased possibility to differentiate into a specific tissue lineage. Furthermore, we investigated if the treatment of hPDLSCs with CBD and MOR could influence differentiation to neuronal lineage, providing a valid tool for stem-based therapy in the neurodegenerative diseases treatment.

## 2. Results

### 2.1. Cytofluorimetric and Morphological Analysis of hPDLSCs

**Figure 1:**
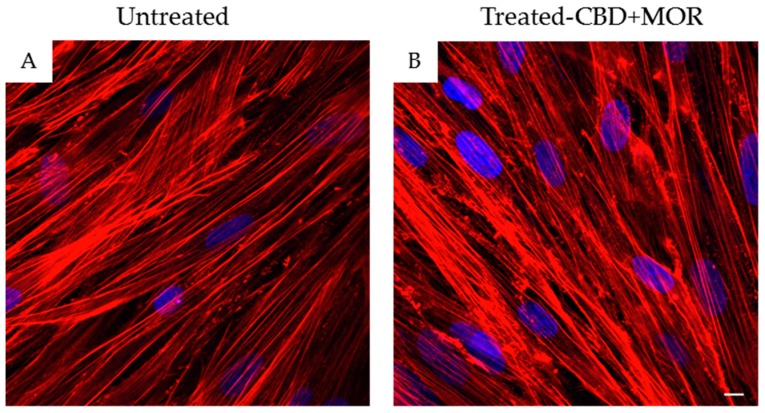
Morphology of hPDLSCs. (**A**) Confocal laser scanning observations of untreated hPDLSCs. (**B**) Confocal laser scanning observations of hPDLSCs treated with the mixture CBD+MOR. Red fluorescence: F-actin; blue fluorescence: nuclei. Scale bar: 5 µm.

In order to confirm hPDLSCs phenotype, flow cytometry analysis was conducted on stem cells at the second passage. Ex vivo expanded hPDLSCs showed positivity for Oct3/4, Sox-2, SSEA-4, CD29, CD44, CD73, CD90, and CD105. On the contrary, the hematopoietic stem cell markers as CD14, CD34, and CD45 were negative (Table 1). Confocal laser morphological observation of untreated hPDLSCs, maintained in standard culture conditions, displayed an elongated spindle-shaped structure (Figure 1A), while hPDLSCs, treated with a mixture of CBD and MOR, showed a cytoskeleton rearrangement, in fact many cells showed a roundish structure (Figure 1B).

### 2.2. Cell Viability Assays

To demonstrate that treatment with CBD and MOR was not toxic for cells and to evaluate cell viability, MTT assay and trypan blue exclusion test were performed.

**Figure 2:**
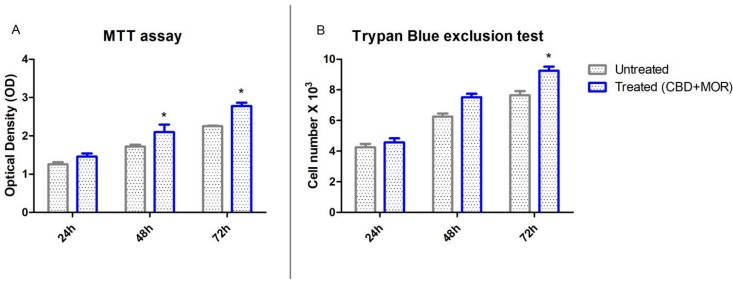
Cell viability assays. (**A**) Viability rate of untreated and treated (CBD+MOR) hPDLSCs at different endpoint (24, 48, and 72 h) evaluated using MTT assay. Data are expressed as the mean of optical density. (**B**) Viability, as determined by Trypan Blue exclusion, of treated and untreated hPDLSCs. Data are expressed as the mean (*n* = 3) number of cells with 95% confidence limits. * *p* < 0.05.

The mixture composed by CBD and MOR showed no toxicity. Figure 2 showed the influence of CBD and MOR on hPDLSCs viability. The obtained results indicated that treated hPDLSCs maintained a viability similar to untreated cells and also showed a slow increase in the growth capacity (Figure 2A). Moreover, trypan blue exclusion test showed the number of the viable treated cells was slowly increased compared with the untreated (Figure 2B).

### 2.3. Immunofluorescence Analysis

In order to evaluate neuronal differentiation of hPDLSCs after combined treatment, we performed immunofluorescence analysis.

**Figure 3:**
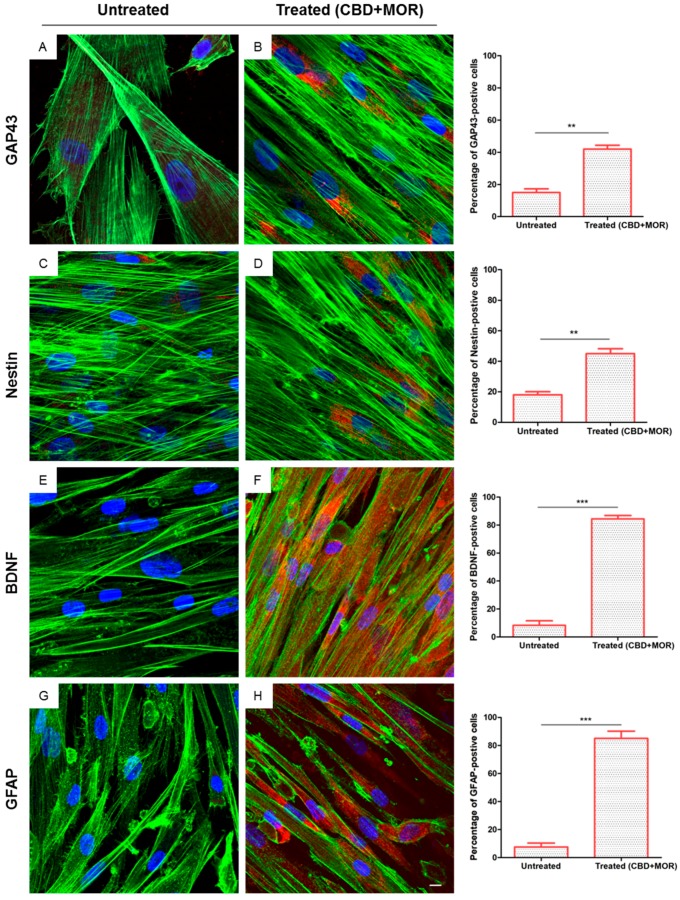
Immunofluorescence analysis. Immunolabeling with GAP43 in (**A**) untreated hPDLSCs and (**B**) treated (CBD+MOR) hPDLSCs. Immunolabeling with neuron-specific NES in (**C**) untreated hPDLSCs and (**D**) treated (CBD+MOR) hPDLSCs. Immunolabeling with neuron-specific BDNF in (**E**) untreated hPDLSCs and (**F**) treated (CBD+MOR) hPDLSCs. Immunolabeling with neuron-specific GFAP in (**G**) untreated hPDLSCs and (**H**) treated (CBD+MOR) hPDLSCs. Histograms represent the percentage of positive cells for the specific markers. ** *p* < 0.01, *** *p* < 0.001 significant difference of hPDLSCs treated with CBD and MOR compared to untreated cells. Green fluorescence: F-actin; red fluorescence: specific markers; blue fluorescence: nuclei. Scale bar: 5 µm.

After 48 h of incubation with CBD+MOR, treated hPDLSCs showed a cytoskeletal remodeling, analyzed through F-actin assembly expression. A qualitative analysis of fluorescent photomicrographs, showed a slow cytoplasmatic expression of GAP43 (growth associated protein 43) and NES (Nestin) in treated hPDLSCs when compared with untreated hPDLSCs, maintained in the same culture conditions (>40%, Figure 3B,D). On the other hand treated hPDLSCs showed a high positivity for BDNF (brain derived neurotrophic factor) and GFAP (glial fibrillary acidic protein), which are well-recognized markers of neuronal and glial cells. As showed in Figure 3F,H, more than 80% of cells were positive for BDNF and GFAP markers.

### 2.4. NGS Analysis

**Figure 4:**
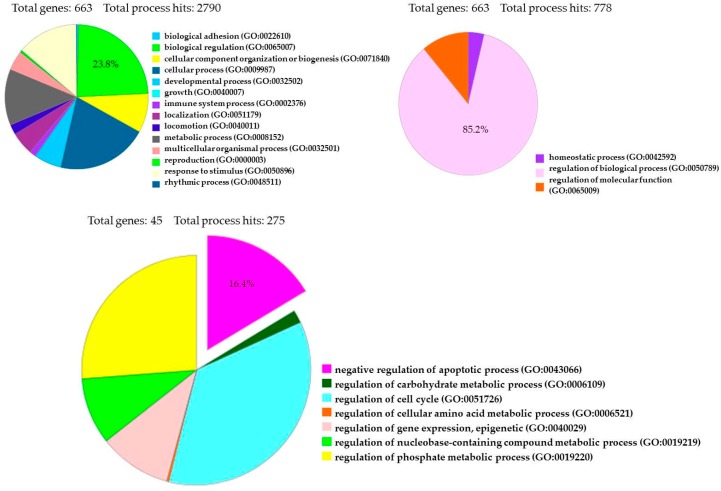
Gene Ontology Analysis of 6843 genes differentially expressed between treated hPDLSCs (MOR+CBD) and untreated cells (CTR).

The transcriptome of treated hPDLSCs (MOR+CBD) and untreated cells (CTR) was carried out using NGS Technology (Illumina, San Diego, CA, USA) and was conducted in triplicate. We identified a total of 6843 genes statistically significant (*q* value < 0.05) and differentially expressed in two experimental groups. More precisely, 3439 genes were upregulated (Log2-fold change between 0.045 and 19.37), while 3404 genes were downregulated (Log2-fold change between −0.055 and −29.32). The fold change indicates the differential gene expression between CTR (untreated-hPDLSCs) and sample (hPDLSCs treated with a combination of MOR and CBD). We investigated the anti-apoptotic effect of treatment with combination of MOR and CBD, by PI3K/Akt/mTOR pathway involvement, through the use of database, such as Gene Ontology and KEGG. Among 6843 genes differentially expressed in our analysis, Gene Ontology identified 663 genes (23.8%) involved in GO: “biological regulation”, among 2790 genes implicated in the regulation of different biological processes, and 663 genes (85.2%) among 778 genes involved in GO: “regulation of biological process”. Moreover, Gene Ontology identified 45 genes (16.4%) involved in GO: “negative regulation of apoptotic process” (Figure 4). Furthermore, Gene Ontology identified 670 genes implicated in GO: “signal transduction” and among them identified 21 genes (3.1%) involved in GO: “PI3 kinase pathway (P00048)”.

The simultaneous consultation of websites as NCBI and KEGG and literature, led us to find a larger number of genes involved in the inhibition of apoptosis (63 genes, Table 2 and Table 3), in death signaling (31 genes, Table 4), in mTOR pathway (63 genes, Table 5), and finally 38 genes belonging to PI3K/Akt/mTOR pathway (Table 6).

In each table, we have reported: gene nomenclature, the whole name of the genes, their expression value (calculated as FPKM, Fragments Per Kilobase of exon model per Million mapped fragments) both in control cells (CTR) and treated cells (MOR+CBD), the corresponding values of Log2-fold change and the statistical significance, indicated by *q* value (FDR, False Discovery Rate) < 0.05. All genes are placed on tables, following descending order of Log2-fold change. Fold change represents the ratio between the value of gene expression in the treated sample compared to the untreated one, calculated as Log2. A positive Log2-fold change value indicates that gene is more expressed in treated sample than in control. A negative value is correlated to a major gene expression in CTR. We used a Log-2 fold change value of −3.5 as cutoff and showed in tables all genes with higher values. In Table 2, we reported all genes involved in “apoptosis inhibition” with a positive value of fold change, while in Table 3 those with negative fold change value. In the table footer of Table 2, main genes showing anti-apoptotic or pro-apoptotic proprieties have been listed.

## 3. Discussion

Stem cell-based therapies for the repair and regeneration of various tissues and organs could provide alternative therapeutic solutions for a number of diseases and neurodegenerative pathologies [20]. hPDLSCs retain all the features of MSCs, such as self-renewal, immunomodulatory, clonogenicity, and multi-tissue differentiation potential [21,22]. Improve and extend survival time of stem cells in transplantation, is one of the main objectives of research in regenerative medicine. A pretreatment with antioxidant, anti-inflammatory, or anticancer compounds can improve their survival and their differentiation capacity. Phytocompounds, such as Cannabidiol and Moringin, were widely studied for their antioxidant [18], anti-inflammatory [16,19], and anticancer [23] proprieties and so a treatment with MOR and CBD of mesenchymal cells can improve their survival. Mesenchymal stem cells can spontaneously differentiate to different cell lineage, showing osteogenic, adipogenic, chondrogenic, and neurogenic differentiation capabilities. We previously demonstrated that a treatment with MOR of hPDLSCs improve their transformation into nervous cell lineage [17]. In this research, we first investigated the effects of combined treatment of CBD and MOR on survival of hPDLSCs and then on neuronal differentiation. Through NGS analysis, we could evaluate the expression of thousands of genes implicated in apoptosis and cell proliferation pathways.

### 3.1. Inhibition of Apoptosis

De novo gene expression and gene rearrangement are necessary to the apoptotic machinery. Although the complete subset of genes that is induced by survival factor is not yet known, several inducible genes, such as *BAX* (BCL2 associated X, apoptosis regulator) and *BAK1* (BCL2 antagonist/killer 1), which are members of pro-apoptotic Bcl-2 family, have been identified as critical components of the apoptotic machinery. Apoptotic process can occur by following extrinsic and intrinsic pathways. The extrinsic pathway is mediated by death receptors such as TNFR (Tumor Necrosis Factor receptors), Fas (Fas cell surface death receptor), and TRAIL (*TNFSF10*, TNF superfamily member 10). Activation of these receptors leads to the recruitment and activation of initiator of caspases cascade (caspases 8 and 10) and finally to activation of an effector caspase, typically caspase 3, that is responsible for the cleavage of a number of death substrates causing membrane blebbing (*LMNA*, lamin A/C and *ROCK1*, Rho associated coiled-coil containing protein kinase 1), and DNA fragmentation (*ACIN1*, apoptotic chromatin condensation inducer 1 and *PARP1*, poly(ADP-ribose) polymerase 1). The intrinsic pathway is largely centered on mitochondria that release cytochrome c, in response to pro-apoptotic members of the Bcl-2 protein family, promoting the formation of the apoptosome, composed of dATP, *APAF1* (apoptosis protease activation factor-1), and activated caspase 9, leading to activation of caspase 3. After cytochrome c release, apoptosis can be still inhibited by IAP (Inhibitor Apoptosis Proteins) family, which binds and inhibits caspases [24]. On the other hand, IAP proteins are inhibited by DIABLO (diablo IAP-binding mitochondrial protein), promoting cell death [25]. Our analysis showed a reduced expression of gene coding for receptor TNFR-1, (*TNFRSF1A*, TNF receptor superfamily member 1A, fold change = −1.32, *q* value = 0.0001) and also transducer of signaling such as TRADD (TRADD, TNFRSF1A associated via death domain, fold change = −0.72, *q* value = 0.0318) are considerably reduced in treated sample. Gene coding for RIP (*RIPK1*, receptor interacting serine/threonine kinase 1, fold change = −0.71, *q* value = 0.0001), a pro-apoptotic protein that enhanced the association between TNFR-1 and TRADD, also had a reduced value of expression, thanks to the high expression value of the *TNFAIP3* gene (TNF alpha induced protein 3, fold change = +2.06, *q* value = 0.0001), coding for A20 protein that exerts an inhibitory activity on RIP. According to Kim et al. (2000), pro-apoptotic C-terminal fragment of RIP inhibited NF-κB (nuclear factor kappa light chain enhancer of activated B cells) activation by suppressing the activity of IKKb (*IKBKB*, I-kB kinase b), which phosphorylates I-Kb (*NFKBIB*, NFKB inhibitor β), an inhibitor of NF-κB [26]. We found that *IKBKB* had a decreasing value of expression (fold change = −0.57, *q* value = 0.0001), while *NFKBIA* (NFKB inhibitor α, fold change = +0.36, *q* value = 0.0025) was increased. Inhibition of NF-κB was confirmed by the reduced value of its expression (*NFKB1*, nuclear factor kappa B subunit 1, fold change = −0.65, *q* value = 0.0001). NF-κB, after translocation into the nucleus, regulates the expression of FLIP protein (*CFLAR*, CASP8, and FADD like apoptosis regulator, fold change = −1.59, *q* value = 0.0001), which is structurally similar to caspase 8 and so is a competitor for FADD (Fas associated via death domain) linkage, inhibiting caspase 8 and apoptosis. Moreover, NF-κB regulates upexpression of *TNFAIP3, BCL2A1* (BCL2 related protein A1, fold change = +14.74, *q* value = 0.0001), *BCLXL* (*BCL2L1*, BCL2-like 1, fold change = +1.94, *q* value = 0.0001), and *XIAP* (X-linked inhibitor of apoptosis, fold change: +0.71, *q* value = 0.0001), belonging to IAP family. *XIAP* is regulated by *DIABLO*, which appears downregulated (fold change= −0.37, *q* value = 0.0018). Caspase 8 (*CASP8*, fold change = −0.903, *q* value = 0.0001), caspase 9 (*CASP9*, fold change = −1.59, *q* value = 0.0002), and caspase 10 (*CASP10*, fold change = −1.55, *q* value = 0.0024) are initiators of caspases cascade, while caspase 6 (*CASP6*, fold change = −1.36, *q* value = 0.0032), caspase 7 (*CASP7*, fold change = −1.98, *q* value = 0.0001), and caspase 3 are effector caspases. Note that all caspases, except caspase 3, that which not appear in our analysis, had negative values of fold change. Following increased expression of pro-apoptotic factors, caspase 9 is activated by apoptosome formation and cytochrome c is released from mitochondrion. *BCL2A1* and *BCL2L1* are anti-apoptotic genes that regulate cytochrome c release from the outer mitochondrial membrane. When *BCL2L1* is over expressed, pores are nonpermeable to pro-apoptotic molecules and the cell survives, while if it is sequestered away by Bim or Bad, proteins such as Bax and Bak are activated and cytochrome c is released, leading to initiation of caspase cascade and apoptotic events. *BAX* gene has a reduced expression in treated cells if compared to untreated cells (fold change = −1.18). Despite this, *BCL2L1* increased and *BAX* and *BAD* (BCL2 associated agonist of cell death, fold change = −0.778, *q* value = 0.0139) decreased in our analysis, the gene coding for cytochrome c (*CYC1*, fold change = +0.66, *q* value = 0.0001) had an increased value of the expression. As mentioned before, also after release of cytochrome c, apoptosis can be still inhibited by over expression of genes coding for inhibitors, such as *XIAP*, *HSPB1* (heat shock protein family B-small-member 1), *HSP90AA1* (heat shock protein 90 alpha family class A member 1), and *HSPA4* (heat shock protein family A (Hsp70) member 4), which showed increased values of fold change. In the mitochondrion, p53 induces Bax and Bak oligomerization and physically interacts with Bcl-xL and Bcl-2, antagonizing their anti-apoptotic effects [27]. In our analysis, we found a decreased value of expression of *TP53* (tumor protein p53, fold change = −1.301, *q* value = 0.0001), coding for p53 protein, confirming the anti-apoptotic effect on hPDLSCs cells of combined treatment. The pro-apoptotic *BAD* gene is also downregulated by Akt (serine/threonine kinase 1). *GSK3* gene can carry out both pro-apoptotic and anti-apoptotic role *GSK3B* (glycogen synthase kinase 3 beta, fold change = +0.310, *q* value = 0.0035) promotes cell death by the mitochondrial intrinsic apoptotic pathway and inhibits the death receptor-mediated extrinsic apoptotic signaling pathway [28] and *GSK3A* (glycogen synthase kinase 3 alpha, fold change = +0.253, *q* value = 0.0231) also plays an important role in the WNT and PI3K signaling pathways.

### 3.2. PI3K/Akt/mTOR Pathway

Akt is also a negative regulator of mTORC1 (mechanistic target of rapamycin complex 1). The mTORC1 complex is composed by the phosphoinositide 3 kinase-related protein kinase (PIKK) mTOR, and the subunits RAPTOR and mLST8 are implicated in cell growth processes such as protein synthesis, lipogenesis, glucose metabolism, proliferation, and survival. Akt inhibits TSC2 (TSC complex subunit 2), a protein associated with hamartin (TSC1) forming a cytosolic complex. This complex negatively regulates mTORC1 signaling, through inhibition of GTPase RHEB, an activator of mTORC1. In our analysis *TSC2* gene (fold change = −0.53, *q* value = 0.0001) is downregulated and *RHEB* (Ras homolog, mTORC1 binding) is upregulated (fold change = +1.62, *q* value = 0.0001). *RHEB* regulates the expression of *MTOR* gene that increases in treated cells (fold change = +0.18, *q* value = 0.0271). The best known substrates of mTORC1 are the eIF4E-binding protein 1 (*EIF4EBP1*, fold change = +0.70, *q* value = 0.0032) and the ribosomal S6 kinase 1 (S6K1) which regulates *RPS6* gene (ribosomal protein S6), coding for ribosomal protein S6. Phosphorylation of EIF4EBP1 destabilizes the 4EBP1–eIF4E complex and activates cap-dependent translation. Also, *RPS6* is upregulated (fold change = +0.076, *q* value = 0.0354) and, together with eIF4E, promotes multiple aspects of protein synthesis. PI3K/Akt and mTOR pathways are related to apoptosis and cell survival and are also largely studied for their activation after cannabinoids administration, through linkage to the CB1 receptor [29,30]. Akt1 upstream regulates cell survival, influencing different biological processes such as apoptosis, through modulation of *BCL2A1*, *BCL2L1*, *BAD*, and *BAX* gene expression; protein synthesis, through activation of genes *EIF4E* and *RPS6*; growth and proliferation, through *SGK1* (serum/glucocorticoid regulated kinase 1, fold change = +4.507, *q* value = 0.0001) activation; cellular glucose metabolism by enhanced expression of *GYS1* (glycogen synthase 1, fold change = +1.022, *q* value = 0.0001); cell cycle progression through upexpression of *MYC* (MYC proto-oncogene, bHLH transcription factor, fold change = +1.89, *q* value = 0.0001), *CCND1* (cyclin D1, fold change = +2.33, *q* value = 0.0001), and *CDK1* (cyclin dependent kinase 1, fold change = +2.64, *q* value = 0.0001). Although genes coding for PI3K (phosphatidylinositol-4,5-bisphosphate 3-kinase catalytic subunit alpha/beta, *PIK3CA* and *PIK3CB*) are downregulated (fold change = −0.514, *q* value = 0.0001; and −0.534, *q* value = 0.0060, respectively) and *AKT* gene (AKT serine/threonine kinase 1) is not present in our analysis, all genes downstream regulated and implicated in cell survival are substantially overexpressed, confirming the activation of PI3K/Akt pathway. Moreover, proteins that negatively regulate AKT signaling pathway, such as PTEN in our analysis was down regulated (*PTEN*, phosphatase, and tensin homolog, fold change = −1.711, *q* value = 0.0001). The PI3K/Akt signaling pathway plays a crucial role also in the survival process of different neuronal cell types as reported by numerous studies [31,32], and its activation is necessary for survival of sympathetic neurons supported by NGF (nerve growth factor), both in the cell body and in the distal axons [33]. Also mesenchymal stem cells, stimulated with BDNF to promote neuronal differentiation, showed an improved survival by apoptosis inhibition, through WNT and PI3K/Akt-dependent signaling pathways [34]. In line with the activation of this pathway, the MTT and Trypan blue assay also showed increased cell viability after the treatment with CBD and MOR. Periodontal ligament stem cells (hPDLSCs), treated with a MOR and CBD combination, showed PI3K/Akt signaling pathway activated and, as previously described, treatment with only MOR stimulated hPDLSCs differentiation to neuronal lineage [17].

### 3.3. Neuronal Differentiation

In immunofluorescent analysis, we observed that treated hPDLSCs had an increased expression of Nestin and GAP43. Nestin is considered a marker of neuronal progenitor cells [35], while the positivity for GAP43 is associated with electrical excitability of stem cells [36]. These results lead us to conclude that the treatment of stem cells with the combination of MOR and CBD activates in vitro neuronal differentiation of totipotent cells.

We have also shown that combined treatment with MOR and CBD leads to an increase in BDNF and the appearance of the neuronal marker GFAP [37].

Also, the cytoskeletal structure of treated cells (CBD+MOR) showed a similar-neuron organization, observed by F-actin labeling (Figure 3).

## 4. Materials and Methods

### 4.1. Ethic Statement

The protocol for isolation and culture of stem cells from human periodontal ligament was approved by the Ethical Committee at the Medical School, “G. d’Annunzio” University, Chieti, Italy (266-17/04/14) and informed consent was given by the patients.

### 4.2. CBD and Moringin Extraction

Pure CBD (>99%) was isolated from Carmagnola, an Italian variety of industrial hemp, provided by greenhouse cultivation (CREA-CIN; Rovigo, Italy), in accordance with their legal status (Authorization SP/106 23/05/2013 of the Ministry of Health, Rome, Italy) following the standardized protocol of the cannabinoid purification to avoid any trace of THC [38,39]. MOR was isolated from powdered seeds of *M. oleifera* seeds (provided by Indena India Pvt. Ltd., Bangalore, India) at the Bologna laboratory (CREA-AA; previously CIN) following methods previously described [40], and the structure was confirmed by NMR (nuclear magnetic resonance) spectroscopic analyses.

### 4.3. hPDLSCs Culture

Periodontal ligament tissue was collected from three healthy patients enrolled in the study, after signing the informed consent, as previously described [41]. hPDLSCs were cultured in MSCGM-CD medium (Lonza, Basel, Switzerland) at 37 °C in a 5% of CO_2_ atmosphere. Cells were treated with the mixture of CBD and MOR at the first passages and when 80% of the confluence was reached. After 48 h of treatment, cells were collected for RNA extraction. Also untreated cells, that were cultured as a control (CTR), were collected at second passage. The experiment was conducted in triplicate. CBD and MOR have been used as a mixture 1:1 (0.5 μM concentration) for a time of incubation of 48 h.

### 4.4. Cytofluorimetric Analysis of hPDLSCs

Evaluation of hPDLSCs phenotype was conducted by flow cytometry, as previously described [17,19]. Briefly, 2.5 × 10^5^ cells were incubated for 30 min with following antibodies: anti-CD44-FITC, anti-CD105-FITC, anti-CD29-PE, and anti-CD45-FITC (Ancell Corporation, Bayport, MN, USA); anti-CD14-FITC (Miltenyi Biotec, Bergisch Gladbach, Germany); OCT3/4-PE, CD73-PE, SOX2-Alexa488, SSEA4-FITC, CD90-FITC (Becton Dickinson, San Jose, CA, USA), and CD34-PE (Beckman Coulter, Fullerton, CA, USA). After incubation with appropriate secondary antibodies, fixation in 1 mL PBS 0.5% paraformaldehyde and washing, cells were analyzed using a FACStar-plus flow cytometry system and the FlowJo™ software v10.0.7 (TreeStar, Ashland, OR, USA).

### 4.5. Cell Viability Assays

In order to evaluate the cellular proliferation and viability, MTT test and Trypan Blue exclusion assay were performed. MTT method is a colorimetric assay based on the use of 3-(4,5-dimethylthiazolyl-2)-2,5-diphenyltetrazoliumbromide solution (Promega, Milan, Italy). MTT assay was performed to assess the effects of the treatment of the mixture (CBD+MOR) on cell proliferation. Treated and untreated 2nd passage hPDLSCs were plated into 96-well plates at a density of 5 × 10^4^ cells per well in 200 μL culture medium. At the designated end points (24, 48, and 72 h) 20 μL PBS MTT solution was added to each well and the plates were incubated at 37 °C for an additional 3 h. The plates were read at 650 nm using a microplate reader (Synergy HT, BioTek Instruments, Winooski, VT, USA) [42]. Moreover, to test the viability of treated and untreated hPDLSCs they were washed with culture medium, incubated for 3 min with trypsin-EDTA to obtain a single cell suspension, and washed with culture medium, and the number of living cells was determined by using Trypan Blue exclusion assay. Experiments have been carried out in triplicates on cells derived from three different donors.

### 4.6. Immunofluorescence Detection

hPDLSCs, at the second passage, treated and untreated with the mixture (CBD and MOR) were fixed and processed for immunofluorescence staining as reported by Diomede et al. [43]. Cells were incubated with mouse primary monoclonal antibody anti-GAP43 1:200 (Sigma Aldrich, Milan, Italy), rabbit anti-Nestin 1:200 (Santa Cruz Biotechnology, Inc., Dallas, TX, USA), rabbit anti-BDNF 1:100 (Santa Cruz Biotechnology), and mouse anti-GFAP 1:100 (Santa Cruz Biotechnology) and mouse anti-GFAP 1:100 (Santa Cruz Biotechnology), followed by anti-mouse Alexa Fluor 488 (Molecular Probes, Life Technologies, Monza, Italy) and anti-rabbit Alexa Fluor 568 (Molecular Probes), respectively.

All samples were incubated with Alexa Fluor 568 phalloidin red fluorescence conjugate (1:400), as a marker of the cytoskeleton actin and with TOPRO to highlight the nuclei [44]. Samples were observed using a CLSM (Zeiss LSM800 META, Zeiss, Jena, Germany). After treatment the percentages of GAP43/Nestin/BDNF/GFAP-positive cells were quantified based on the 15 images collected randomly. Experiments have been carried out in triplicates on cells derived from three different donors.

### 4.7. RNA Libraries Preparation for NGS Analysis

Experiments have been carried out in triplicates on cells derived from three different donors. Total RNA extraction was performed using Reliaprep RNA Cell Miniprep System (Promega Corporation, Madison, WI, USA) and quantified by QuantiFluor^®^ RNA System kit and Quantus Fluorometer (Promega). Sequencing was done in triplicate. As previously described [17], and in accordance with TruSeq RNA Access library kit protocol (Illumina, San Diego, CA, USA), about 50 ng of total RNA has been fragmented at 94 °C for 8 min, obtaining fragments >200 nt. Using SuperScript II reverse transcriptase (Invitrogen, Carlsbad, CA, USA) a first strand of cDNA has been synthesized. The complementary strand of cDNA has been synthesized thanks to Second Strand Marking Master Mix. Two successive steps of adapter hybridization/PCR amplification are necessary for the identification of each sample and ligation to the flow cell. The first reaction of hybridization uses exome capture probes in order to select and to enrich specific cDNA library regions. A quantity of 200 ng of resulting indexed library was used as input of a second hybridization/PCR amplification. Finally, the library has been qualitatively validated using a Bioanalyzer instrument (Agilent High Sensitivity DNA Kit, Richardson, TX, USA) and quantified using QuantiFluor^®^ ONE dsDNA System kit and Quantus Fluorometer (Promega).

The obtained library has been denatured by adding 2 M NaOH and diluted until final concentration of 14 pM. MiSeq Reagent Kit v3 (150 cycles) has been used for sequencing on the MiSeq Instrument (Illumina, San Diego, CA, USA).

### 4.8. Data Analysis

The CASAVA software version 1.8 (Illumina, San Diego, CA, USA) was used to generate the “Fastq.File”. The fastq.files have been aligned to the “homo sapiens UCSC hg19” reference sequence, using the STAR, Open Source software (software distributed under GPLv3 license and can be downloaded from http://code.google.com/p/rna-star/). For statistical analysis, we used Cufflinks Assembly & DE package version 2.0.0. (software of Illumina propriety; cole-trapnell-lab.github.io/cufflinks/), in order to evaluate the statistically relevant data. Gene expression values have been calculated as FPKM (fragments per kilobase of exon per million fragments mapped) by using the Cufflinks algorithm, while differential gene expression values between the two different experimental groups (treated and untreated hPDLSCs) have been calculated using Cuffdiff algorithm. To normalize and to compare all samples, the FPKM value was calculated by applying the mathematical formula: (1000× read count)/(number of gene covered bases × number of mapped fragments in million). Only statistically significant data with *q* value (FDR, False Discovery Rate) <0.05 have been considered for the analysis. A scatter plot of the Log2 of the FPKM was used in order to compare the two experimental groups. The NCBI website (http://www.ncbi.nlm.nih.gov/gene), Gene Ontology (http://www.geneontology.org/), and KEGG database (http://www.genome.jp/kegg/) have been used to investigate and classify the differentially expressed genes between the two experimental groups, related to cellular processes such as: cell division, proliferation, and regulation of apoptosis. In particular, we focused on involvement of PI3K/Akt/mTOR pathway in apoptosis inhibition, by upregulation of anti-apoptotic genes and contemporary downregulation of pro-apoptotic ones.

## 5. Conclusions

Stem cells represent an encouraging perspective for regenerative medicine and periodontal ligament MSCs retain self-renewal, clonogenicity, and multi-tissue differentiation potential. The possibility to elongate their survival is essential to the success of stem cell-based therapy. A pretreatment of stem cells with phytocompounds could improve their survival in the re-implanted tissue and their differentiation capacity. Through NGS transcriptome analysis, we demonstrated that hPDLSCs treated with a combination of MOR and CBD showed improved survival characteristics compared to untreated cells. Prolonged survival is accompanied by inhibition of apoptosis, through activation of the PI3K/Akt/mTOR pathway, as demonstrated by differential gene expression between treated and untreated cells. Moreover, we demonstrated that hPDLSCs treated with MOR and CBD for 48 h showed an increased expression of the neuronal progenitor cell marker Nestin, and of the neuronal markers GAP43, GFAP, and BDNF evaluated by immunofluorescence analysis, confirming an improved neurogenic differentiation. However, we only performed in vitro studies, for this reason in vivo models are needed to evaluate the effects of CBD and MOR on MSCs after transplantation in animals.

## Figures and Tables

**Table 1:** Cytofluorimetric analysis of hPDLSCs. In the table, were reported: cell typology, specific markers, and percentage of expression of each marker. All markers of stem cells and mesenchymal stem cells were expressed on the surface, while hematopoietic cell markers were not expressed.

Cell Lineage	Markers	% Of Expression
Stem cells	Oct3/4	94.3 ± 2.2%
	Sox-2	96.4 ± 0.7%
	SSEA-4	96.4 ± 2.5%
MSCs	CD29	97.8 ± 2.4%
	CD44	95.2 ± 3.3%
	CD73	96.3 ± 1.1%
	CD90	95.2 ± 3.1%
	CD105	96.3 ± 3.2%
Hematopoietic	CD14	ND
	Cd34	ND
	CD45	ND

**Table 2:** Upregulated genes involved in “Inhibition of Apoptosis”.

Gene	Name	CTR	MOR+CBD	Log2-FoldChange	*Q* Value
*BCL2A1*	BCL2 related protein A1	0.0001	2.7356	14.739	0.000108528
*PRPS1*	phosphoribosyl pyrophosphate synthetase 1	12.773	82.505	2.691	0.000108528
*CDK1*	cyclin dependent kinase 1	1.601	9.994	2.642	0.000108528
*PRKAG2*	protein kinase AMP-activated non-catalytic subunit gamma 2	3.731	16.269	2.124	0.000108528
*CYCS*	cytochrome c, somatic	4.446	18.7684	2.077	0.000108528
*TNFAIP3*	TNF alpha induced protein 3	0.81318	3.40641	2.066	0.000108528
*BCL2L1*	BCL2 like 1	2.45308	9.41279	1.940	0.000108528
*CDC37*	cell division cycle 37	51.8182	194.202	1.906	0.000108528
*CRADD*	CASP2 and RIPK1 domain containing adaptor with death domain	4.94457	11.2444	1.185	0.000679197
*HSPB1*	heat shock protein family B (small) member 1	279.982	688.126	1.297	0.000108528
*HSP90AA1*	heat shock protein 90 alpha family class A member 1	271.592	621.726	1.195	0.000108528
*PMAIP1*	phorbol-12-myristate-13-acetate-induced protein 1	1.755	3.424	0.964	0.0248542
*MAPK1*	mitogen-activated protein kinase 1	37.1635	61.9931	0.738	0.000108528
*XIAP*	X-linked inhibitor of apoptosis	6.18581	10.1329	0.712	0.000108528
*CYC1*	cytochrome c1	27.7517	43.9185	0.662	0.000108528
*HSPA4*	heat shock protein family A (Hsp70) member 4	59.0696	88.7283	0.586	0.000108528
*CYLD*	CYLD lysine 63 deubiquitinase	11.8515	17.1827	0.536	0.000108528
*MAPK3*	mitogen-activated protein kinase 3	43.4258	61.3592	0.499	0.000108528
*CASP2*	Caspase 2	6.65398	9.33578	0.488	0.000679197
*ROCK1*	Rho associated coiled-coil containing protein kinase 1	15.711	20.961	0.416	0.000108528
*MAP3K5*	mitogen-activated protein kinase kinase kinase 5	4.264	5.599	0.393	0.0185603
*NFKBIA*	NFKB inhibitor alpha	30.3404	38.8837	0.358	0.00254092
*AIFM1*	apoptosis inducing factor mitochondria associated 1	22.697	28.454	0.3579	0.00342361
*GSK3B*	glycogen synthase kinase 3 beta	7.29297	9.04263	0.310	0.00350034
*PRKAG1*	protein kinase AMP-activated non-catalytic subunit gamma 1	22.7303	27.4994	0.275	0.0360551
*GSK3A*	glycogen synthase kinase 3 alpha	24.6326	29.3496	0.253	0.0231286

In the table, we have reported for each gene the expression value, calculated as FPKM (Fragments per Kilobase of exon model per Million mapped fragments), both in untreated (CTR) and treated cells (MOR+CBD), Log 2-fold change positive values (upregulated genes), and a *q*-value (FDR) < 0.05 (statistical significance). Anti-apoptotic genes: *BCL2A1*, *TNFAIP3*, *BCL2L1*, *HSPB1*, *HSP90AA1*, *MAPK1*, *XIAP*, *GSK3A/B*, *MCL1*, *BIRC2*, *TP53*, *CFLAR*; Pro-apoptotic genes: *CYCS*, *CYC1*, *CRADD*, *CASP2*, *GSK3A/B*, *DIABLO*, *APAF1*, *IKBKB*, *NFKB1*, *RIPK1*, *BAD*, *CASP8*, *HTRA2*, *BAX*, *CASP6*, *CASP10*, *CASP9*, *CASP8.*

**Table 3:** Downregulated genes involved in “Inhibition of Apoptosis”.

Gene	Name	CTR	MOR+CBD	Log2-FoldChange	*Q* Value
*MCL1*	MCL1, BCL2 family apoptosis regulator	15.5899	13.3424	−0.224	0.0365845
*PRKACA*	protein kinase cAMP-activated catalytic subunit alpha	36.0344	29.1389	−0.306	0.000769756
*JUN*	Jun proto-oncogene, AP-1 transcription factor subunit	45.231	35.688	−0.341	0.000108528
*BRAF*	B-Raf proto-oncogene, serine/threonine kinase	18.1853	14.3317	−0.343	0.00374229
*DIABLO*	diablo IAP-binding mitochondrial protein	24.9695	19.2303	−0.377	0.0018832
*APAF1*	apoptotic peptidase activating factor 1	5.49731	4.023	−0.450	0.00146168
*NRAS*	proto-oncogene, GTPase	9.22853	6.45952	−0.514	0.000209655
*PIK3CA*	phosphatidylinositol-4,5-bisphosphate 3-kinase catalytic subunit alpha	17.6961	12.3847	−0.515	0.000108528
*PIK3CB*	phosphatidylinositol-4,5-bisphosphate 3-kinase catalytic subunit beta	3.9361	2.71759	−0.534	0.00600177
*IKBKB*	inhibitor of nuclear factor kappa B kinase subunit beta	9.88765	6.66404	−0.569	0.000108528
*PIK3CD*	phosphatidylinositol-4,5-bisphosphate 3-kinase catalytic subunit delta	11.4445	7.6164	−0.587	0.000108528
*APP*	amyloid beta precursor protein	812.971	538.076	−0.595	0.000108528
*NFKB1*	nuclear factor kappa B subunit 1	9.63897	6.14856	−0.648	0.000108528
*RIPK1*	receptor interacting serine/threonine kinase 1	8.97176	5.47512	−0.712	0.000108528
*JAK2*	Janus kinase 2	3.17143	1.92764	−0.718	0.00382528
*ROCK2*	Rho associated coiled-coil containing protein kinase 2	25.266	15.313	−0.722	0.000108528
*TRADD*	TNFRSF1A associated via death domain	6.42414	3.88442	−0.726	0.0318132
*BAD*	BCL2 associated agonist of cell death	12.1113	7.0611	−0.778	0.0138987
*STAT1*	signal transducer and activator of transcription 1	220.682	120.078	−0.878	0.000108528
*STAT3*	signal transducer and activator of transcription 3	60.0522	32.1874	−0.900	0.000108528
*CASP8*	caspase 8	14.8563	7.94494	−0.903	0.000108528
*BIRC2*	baculoviral IAP repeat containing 2	23.7492	11.4038	−1.058	0.000108528
*CHEK2*	checkpoint kinase 2	9.375	4.444	−1.077	0.000209655
*PIDD1*	p53-induced death domain protein 1	2.819	1.274	−1.145	0.00453657
*IKBKG*	inhibitor of nuclear factor kappa B kinase subunit gamma	20.3793	9.20785	−1.146	0.000108528
*HTRA2*	HtrA serine peptidase 2	16.330	7.278	−1.166	0.000108528
*BAX*	BCL2 associated X, apoptosis regulator	72.4174	31.9437	−1.181	0.000108528
*ATM*	ATM serine/threonine kinase	17.969	7.731	−1.216	0.000108528
*TP53*	tumor protein p53	21.8171	8.8547	−1.301	0.000108528
*TNFRSF1A*	TNF receptor superfamily member 1A	130.525	52.1762	−1.323	0.000108528
*CASP6*	Caspase 6	5.42611	2.11284	−1.361	0.00318817
*CASP10*	Caspase 10	2.91088	0.99445	−1.5495	0.00245968
*CASP9*	Caspase 9	3.91636	1.29947	−1.591	0.000209655
*CFLAR*	CASP8 and FADD like apoptosis regulator	35.1508	11.6351	−1.595	0.000108528
*PTEN*	phosphatase and tensin homolog	50.3299	15.3705	−1.711	0.000108528
*CASP7*	Caspase 7	11.4138	2.89071	−1.981	0.000108528

In the table, we have reported for each gene the expression value, calculated as FPKM (Fragments Per Kilobase of exon model per Million mapped fragments), both in untreated (CTR) and treated cells (MOR+CBD), Log 2-fold change negative values (downregulated genes), and a *q*-value (FDR) < 0.05 (statistical significance).

**Table 4:** Up- and downregulated genes, involved in the “Cell death receptor signaling” pathway.

Gene	Name	CTR	MOR+CBD	Log2-Fold Change	*Q* Value
*TNFRSF10A*	TNF receptor superfamily member 10a	3.73692	10.4229	1.47983	0.000108528
*MAP2K1*	mitogen-activated protein kinase kinase 1	25.1462	50.4431	1.00432	0.000108528
*XIAP*	X-linked inhibitor of apoptosis	6.18581	10.1329	0.71202	0.000108528
*CYLD*	CYLD lysine 63 deubiquitinase	11.8515	17.1827	0.53589	0.000108528
*CHUK*	conserved helix-loop-helix ubiquitous kinase	11.3559	15.2974	0.42985	0.000494671
*ROCK1*	Rho associated coiled-coil containing protein kinase 1	15.7113	20.9609	0.4159	0.000108528
*RELA*	RELA proto-oncogene, NF-kB subunit	18.2587	24.1675	0.40449	0.00146168
*MAP3K5*	mitogen-activated protein kinase kinase kinase 5	4.26421	5.59967	0.39306	0.0185603
*NFKBIA*	NFKB inhibitor alpha	30.3404	38.8837	0.35793	0.00254092
*MPRIP*	myosin phosphatase Rho interacting protein	21.5258	18.5193	−0.217	0.000108528
*PARP1*	poly(ADP-ribose) polymerase 1	45.8356	39.1245	−0.2284	0.000494671
*ACIN1*	apoptotic chromatin condensation inducer 1	30.3285	25.0361	−0.2767	0.000494671
*DIABLO*	diablo IAP-binding mitochondrial protein	24.9695	19.2303	−0.3768	0.0018832
*APAF1*	apoptotic peptidase activating factor 1	5.49731	4.023	−0.4505	0.00146168
*RPAIN*	RPA interacting protein	12.2724	8.85997	−0.47	0.0253385
*FAS*	Fas cell surface death receptor	48.1152	33.0363	−0.5424	0.000108528
*IKBKB*	inhibitor of nuclear factor kappa B kinase subunit beta	9.88765	6.66404	−0.5692	0.000108528
*RIPK1*	receptor interacting serine/threonine kinase 1	8.97176	5.47512	−0.7125	0.000108528
*TRADD*	TNFRSF1A associated via death domain	6.42414	3.88442	−0.7258	0.0318132
*MAP4K4*	mitogen-activated protein kinase kinase kinase kinase 4	128.313	75.8702	−0.7581	0.000108528
*CASP8*	caspase 8	14.8563	7.94494	−0.903	0.000108528
*BIRC2*	baculoviral IAP repeat containing 2	23.7492	11.4038	−1.0584	0.000108528
*IKBKG*	inhibitor of nuclear factor kappa B kinase subunit gamma	20.3793	9.20785	−1.1462	0.000108528
*TNFRSF10B*	TNF receptor superfamily member 10b	94.0163	40.7967	−1.2045	0.000108528
*LMNA*	lamin A/C	1373.63	587.557	−1.2252	0.000108528
*TNFRSF1A*	TNF receptor superfamily member 1A	130.525	52.1762	−1.3229	0.000108528
*CASP6*	Caspase 6	5.42611	2.11284	−1.3607	0.00318817
*CASP10*	Caspase 10	2.91088	0.99445	−1.5495	0.00245968
*CASP9*	Caspase 9	3.91636	1.29947	−1.5916	0.000209655
*CFLAR*	CASP8 and FADD like apoptosis regulator	35.1508	11.6351	−1.5951	0.000108528
*CASP7*	Caspase 7	11.4138	2.89071	−1.9813	0.000108528

In the table, we have reported: the nomenclature, the whole name, the value of gene expression (FPKM) in both untreated (CTR) and treated cells (MOR+CBD), Log2-fold change values, and *q* values (FDR) < 0.05.

**Table 5:** Up- and downregulated genes, involved in “mTOR pathway”.

Gene	Name	CTR	MOR+CBD	Log2-Fold Change	*Q* Value
*SGK1*	serum/glucocorticoid regulated kinase 1	4.69263	106.74	4.50757	0.000108528
*CDC37*	cell division cycle 37	51.8182	194.202	1.90603	0.000108528
*TMX2*	thioredoxin related transmembrane protein 2	25.587	84.3633	1.72121	0.000108528
*BIRC5*	Baculoviral IAP repeat containing 5	0.876953	2.84342	1.69706	0.00508584
*RHEB*	Ras homolog, mTORC1 binding	15.1136	46.575	1.62371	0.000108528
*GK*	glycerol kinase	5.78668	13.9776	1.27231	0.000108528
*YWHAH*	tyrosine3-monooxygenase/tryptophan 5-monooxygenase activation protein beta	15.6913	35.0565	1.15972	0.000108528
*NCL*	nucleolin	178.099	377.828	1.08505	0.000108528
*UBE2D1*	ubiquitin conjugating enzyme E2 D1	4.46588	8.44974	0.919964	0.000108528
*AKT1S1*	AKT1 substrate 1	5.66925	9.57204	0.755668	0.00262175
*TSC1*	TSC complex subunit 1	7.62739	12.8277	0.75	0.000108528
*MAPK1*	mitogen-activated protein kinase 1	37.1635	61.9931	0.73822	0.000108528
*GRB2*	growth factor receptor bound protein 2	17.0713	28.1494	0.721528	0.000108528
*XIAP*	X-linked inhibitor of apoptosis	6.18581	10.1329	0.712017	0.000108528
*EIF4EBP1*	eukaryotic translation initiation factor 4E binding protein 1	14.858	24.2207	0.705002	0.00318817
*CSK*	C-terminal Src kinase	6.02989	9.54202	0.662163	0.00262175
*NDUFA6*	NADH:ubiquinone oxidoreductase subunit A6	44.3725	67.7367	0.610273	0.000108528
*VRK1*	vaccinia related kinase 1	11.1566	16.9088	0.599877	0.00229935
*IDH3A*	isocitrate dehydrogenase 3 (NAD(+)) alpha	6.79514	10.2507	0.593141	0.00245968
*RICTOR*	RPTOR independent companion of MTOR complex 2	4.49855	6.77801	0.591403	0.000108528
*IDH3G*	isocitrate dehydrogenase 3 (NAD(+)) gamma	24.8055	36.6474	0.56305	0.000108528
*RPS7*	ribosomal protein S7	1220.66	1745.62	0.516083	0.000108528
*MAPK3*	mitogen-activated protein kinase 3	43.4258	61.3592	0.498727	0.000108528
*NAA10*	N(alpha)-acetyltransferase 10, NatA catalytic subunit	65.211	90.1417	0.467079	0.000108528
*MAPKAP1*	mitogen-activated protein kinase associated protein 1	24.7763	34.1371	0.462382	0.000108528
*PPP1CA*	protein phosphatase 1 catalytic subunit alpha	69.6542	95.9494	0.462064	0.000108528
*UBC*	ubiquitin C	643.861	871.181	0.436224	0.000108528
*CHUK*	conserved helix-loop-helix ubiquitous kinase	11.3559	15.2974	0.429848	0.000494671
*RELA*	RELA proto-oncogene, NF-kB subunit	18.2587	24.1675	0.404486	0.00146168
*ATP1A1*	ATPase Na+/K+ transporting subunit alpha 1	107.718	138.718	0.364897	0.000108528
*MYL12B*	myosin light chain 12B	225.236	289.645	0.362849	0.000108528
*NFKBIA*	NFKB inhibitor alpha	30.3404	38.8837	0.357928	0.00254092
*GSK3B*	glycogen synthase kinase 3 beta	7.29297	9.04263	0.310236	0.00350034
*PRKAA1*	protein kinase AMP-activated catalytic subunit alpha 1	10.8385	12.9494	0.256722	0.000769756
*SDCBP*	Syndecan binding protein	98.2519	112.121	0.190497	0.000494671
*MTOR*	mechanistic target of rapamycin kinase	10.8825	12.325	0.179576	0.0270992
*CDKN1A*	cyclin dependent kinase inhibitor 1A	189.675	171.028	−0.14929	
*YWHAB*	tyrosine 3-monooxygenase/tryptophan 5-monooxygenase activation protein beta	36.9941	31.2586	−0.24304	0.00270215
*IRS1*	insulin receptor substrate 1	18.1379	15.1972	−0.25521	0.000108528
*BRAF*	B-Raf proto-oncogene, serine/threonine kinase	18.1853	14.3317	−0.34356	0.00374229
*ITGB1*	integrin subunit beta 1	659.512	498.137	−0.40486	0.000108528
*SOS2*	SOS Ras/Rho guanine nucleotide exchange factor 2	7.55781	5.65783	−0.41772	0.00294683
*APAF1*	apoptotic peptidase activating factor 1	5.49731	4.023	−0.45045	0.00146168
*ULK1*	unc-51 like autophagy activating kinase 1	4.66539	3.33227	−0.48549	0.0102621
*PIK3CA*	phosphatidylinositol-4,5-bisphosphate 3-kinase catalytic subunit alpha	17.6961	12.3847	−0.5149	0.000108528
*NRAS*	NRAS proto-oncogene, GTPase	9.22853	6.45952	−0.51467	0.000209655
*TSC2*	TSC complex subunit 2	37.2339	25.8014	−0.52917	0.000108528
*RPS6KB1*	ribosomal protein S6 kinase B1	7.68103	5.28824	−0.53851	0.000306871
*IKBKB*	inhibitor of nuclear factor kappa B kinase subunit beta	9.88765	6.66404	−0.56923	0.000108528
*RPS6KA3*	ribosomal protein S6 kinase A3	12.1111	7.90192	−0.61606	0.000108528
*PRDX4*	peroxiredoxin 4	141.666	91.3201	−0.63349	0.000108528
*GRB10*	growth factor receptor bound protein 10	5.92727	3.55434	−0.73779	0.000209655
*RHOD*	ras homolog family member D	8.51073	4.80959	−0.82337	0.0175082
*CLIP1*	CAP-Gly domain containing linker protein 1	24.6133	13.7533	−0.83966	0.000108528
*MAP2K7*	mitogen-activated protein kinase kinase 7	4.97253	2.44847	−1.0221	0.000402551
*SOS1*	SOS Ras/Rac guanine nucleotide exchange factor	26.454	12.3817	−1.09527	0.000108528
*IKBKG*	inhibitor of nuclear factor kappa B kinase subunit gamma	20.3793	9.20785	−1.14617	0.000108528
*APBB3*	amyloid beta precursor protein binding family B member 3	3.31305	1.35579	−1.28903	0.00893961
*TP53*	tumor protein p53	21.8171	8.8547	−1.30095	0.000108528
*NFIL3*	nuclear factor, interleukin 3 regulated	7.77916	3.03723	−1.35686	0.000108528
*PTEN*	phosphatase and tensin homolog	50.3299	15.3705	−1.71126	0.000108528
*DDIT4*	DNA damage inducible transcript 4	18.464	3.98013	−2.21383	0.000108528
*WNT5A*	Wnt family member 5A	69.3887	8.13031	−3.09332	0.000108528

In table, we have reported: the nomenclature, the whole name, the gene expression value (FPKM) in untreated (CTR) and treated cells (MOR+CBD), Log2-fold change values, and *q* values (FDR) < 0.05.

**Table 6:** Up- and downregulated genes, involved in “PI3K/Akt/mTOR pathway”.

Gene	Name	CTR	MOR+CBD	Log2-Fold Change	*Q* Value
*BCL2A1*	BCL2 related protein A1	0.0001	2.73565	14.73959	0.000108528
*SGK1*	serum/glucocorticoid regulated kinase 1	4.69263	106.74	4.50757	0.000108528
*CDK1*	cyclin dependent kinase 1	1.60127	9.99442	2.64191	0.000108528
*CCND1*	cyclin D1	57.5334	289.455	2.33087	0.000108528
*BCL2L1*	BCL2 like 1	2.45308	9.41279	1.94003	0.000108528
*MYC*	MYC proto-oncogene, bHLH transcription factor	5.20354	19.3543	1.89509	0.000108528
*RHEB*	Ras homolog, mTORC1 binding	15.1136	46.575	1.62371	0.000108528
*BRCA1*	BRCA1, DNA repair associated	1.39172	3.87502	1.47734	0.000108528
*GYS1*	glycogen synthase 1	20.1343	40.8951	1.02227	0.000108528
*CRTC2*	CREB regulated transcription coactivator 2	7.74262	14.2376	0.87881	0.000108528
*AKT1S1*	AKT1 substrate 1	5.66925	9.57204	0.755668	0.00262175
*TSC1*	TSC complex subunit 1	7.62739	12.8277	0.75	0.000108528
*MAPK1*	mitogen-activated protein kinase 1	37.1635	61.9931	0.73822	0.000108528
*EIF4EBP1*	eukaryotic translation initiation factor 4E binding protein 1	14.858	24.2207	0.705002	0.00318817
*RPS6KB2*	ribosomal protein S6 kinase B2	10.6792	16.799	0.65357	0.00129168
*CREB3*	cAMP responsive element binding protein 3	15.8771	24.3326	0.615947	0.000108528
*EIF4E*	eukaryotic translation initiation factor 4E	13.0091	17.7561	0.448797	0.000108528
*NFKBIA*	NFKB inhibitor alpha	30.3404	38.8837	0.357928	0.00254092
*GSK3B*	glycogen synthase kinase 3 beta	7.29297	9.04263	0.310236	0.00350034
*PRKAA1*	protein kinase AMP-activated catalytic subunit alpha 1	10.8385	12.9494	0.256722	0.0156024
*GSK3A*	glycogen synthase kinase 3 alpha	24.6326	29.3496	0.252774	0.0231286
*MTOR*	mechanistic target of rapamycin kinase	10.8825	12.325	0.179576	0.0270992
*RPS6*	ribosomal protein S6	759.592	800.781	0.0761832	0.0354641
*MCL1*	MCL1, BCL2 family apoptosis regulator	15.5899	13.3424	−0.224596	0.0365845
*BRAF*	B-Raf proto-oncogene, serine/threonine kinase	18.1853	14.3317	−0.343559	0.00374229
*EIF4B*	eukaryotic translation initiation factor 4B	108.227	84.7943	−0.352014	0.000108528
*PIK3CA*	phosphatidylinositol-4,5-bisphosphate 3-kinase catalytic subunit alpha	17.6961	12.3847	−0.514879	0.000108528
*TSC2*	TSC complex subunit 2	37.2339	25.8014	−0.529167	0.000108528
*PIK3CB*	phosphatidylinositol-4,5-bisphosphate 3-kinase catalytic subunit beta	3.9361	2.71759	−0.534435	0.00600177
*RPS6KB1*	ribosomal protein S6 kinase B1	7.68103	5.28824	−0.538514	0.000306871
*IKBKB*	inhibitor of nuclear factor kappa B kinase subunit beta	9.88765	6.66404	−0.569232	0.000108528
*NFKB1*	nuclear factor kappa B subunit 1	9.63897	6.14856	−0.64863	0.000108528
*RBL2*	RB transcriptional corepressor like 2	12.9876	7.44702	−0.802397	0.000108528
*MAP2K7*	mitogen-activated protein kinase kinase 7	4.97253	2.44847	−1.0221	0.000402551
*TP53*	tumor protein p53	21.8171	8.8547	−1.30095	0.000108528
*PCK2*	phosphoenolpyruvate carboxykinase 2, mitochondrial	10.9312	4.17877	−1.38731	0.000108528
*CASP9*	Caspase 9	3.91636	1.29947	−1.59159	0.000209655
*PTEN*	phosphatase and tensin homolog	50.3299	15.3705	−1.71126	0.000108528

In table, we have reported: the nomenclature, the whole name, the gene expression values in untreated (CTR) or treated cells (MOR+CBD), Log2-fold change values, and *q* values (FDR) < 0.05.

## Abbreviations

hPDLSCs	Human Periodontal Ligament Stem Cells
MOR	Moringin
CBD	Cannabidiol
NGS	Next Generation Sequencing
MSCs	Mesenchymal Stem Cells
CTR	Control
MTT	3-(4,5-dimethylthiazolyl-2)-2,5-diphenyltetrazolium bromide

## References

[1] Hass R., Kasper C., Bohm S., Jacobs R. (2011). Different populations and sources of human mesenchymal stem cells (MSC): A comparison of adult and neonatal tissue-derived MSC. Cell Commun. Signal..

[2] Sonoyama W., Liu Y., Yamaza T., Tuan R.S., Wang S., Shi S., Huang G.T. (2008). Characterization of the apical papilla and its residing stem cells from human immature permanent teeth: A pilot study. J. Endod..

[3] Gronthos S., Brahim J., Li W., Fisher L.W., Cherman N., Boyde A., DenBesten P., Robey P.G., Shi S. (2002). Stem cell properties of human dental pulp stem cells. J. Dent. Res..

[4] Miura M., Gronthos S., Zhao M., Lu B., Fisher L.W., Robey P.G., Shi S. (2003). Shed: Stem cells from human exfoliated deciduous teeth. Proc. Natl. Acad. Sci. USA.

[5] Huang G.T., Gronthos S., Shi S. (2009). Mesenchymal stem cells derived from dental tissues vs. Those from other sources: Their biology and role in regenerative medicine. J. Dent. Res..

[6] Trubiani O., Giacoppo S., Ballerini P., Diomede F., Piattelli A., Bramanti P., Mazzon E. (2016). Alternative source of stem cells derived from human periodontal ligament: A new treatment for experimental autoimmune encephalomyelitis. Stem Cell Res. Ther..

[7] Gugliandolo A., Diomede F., Cardelli P., Bramanti A., Scionti D., Bramanti P., Trubiani O., Mazzon E. (2018). Transcriptomic analysis of gingival mesenchymal stem cells cultured on 3D bioprinted scaffold: A promising strategy for neuroregeneration. J. Biomed. Mater. Res. Part A.

[8] Diomede F., Caputi S., Merciaro I., Frisone S., D’Arcangelo C., Piattelli A., Trubiani O. (2014). Pro-inflammatory cytokine release and cell growth inhibition in primary human oral cells after exposure to endodontic sealer. Int. Endod. J..

[9] Diomede F., Rajan T.S., Gatta V., D’Aurora M., Merciaro I., Marchisio M., Muttini A., Caputi S., Bramanti P., Mazzon E. (2017). Stemness maintenance properties in human oral stem cells after long-term passage. Stem Cells Int..

[10] Granero-Molto F., Weis J.A., Longobardi L., Spagnoli A. (2008). Role of mesenchymal stem cells in regenerative medicine: Application to bone and cartilage repair. Expert Opin. Biol. Ther..

[11] Richardson S.M., Kalamegam G., Pushparaj P.N., Matta C., Memic A., Khademhosseini A., Mobasheri R., Poletti F.L., Hoyland J.A., Mobasheri A. (2016). Mesenchymal stem cells in regenerative medicine: Focus on articular cartilage and intervertebral disc regeneration. Methods.

[12] Ballerini P., Diomede F., Petragnani N., Cicchitti S., Merciaro I., Cavalcanti M., Trubiani O. (2017). Conditioned medium from relapsing-remitting multiple sclerosis patients reduces the expression and release of inflammatory cytokines induced by lps-gingivalis in THP-1 and MO3.13 cell lines. Cytokine.

[13] Giacoppo S., Thangavelu S.R., Diomede F., Bramanti P., Conti P., Trubiani O., Mazzon E. (2017). Anti-inflammatory effects of hypoxia-preconditioned human periodontal ligament cell secretome in an experimental model of multiple sclerosis: A key role of IL-37. FASEB J..

[14] Galuppo M., Giacoppo S., De Nicola G.R., Iori R., Navarra M., Lombardo G.E., Bramanti P., Mazzon E. (2014). Antiinflammatory activity of glucomoringin isothiocyanate in a mouse model of experimental autoimmune encephalomyelitis. Fitoterapia.

[15] Giacoppo S., Iori R., Bramanti P., Mazzon E. (2017). Topical moringin-cream relieves neuropathic pain by suppression of inflammatory pathway and voltage-gated ion channels in murine model of multiple sclerosis. Mol. Pain.

[16] Giacoppo S., Rajan T.S., Iori R., Rollin P., Bramanti P., Mazzon E. (2017). The alpha-cyclodextrin complex of the moringa isothiocyanate suppresses lipopolysaccharide-induced inflammation in raw 264.7 macrophage cells through AKT and p38 inhibition. Inflamm. Res..

[17] Romeo L., Diomede F., Gugliandolo A., Scionti D., Lo Giudice F., Lanza Cariccio V., Iori R., Bramanti P., Trubiani O., Mazzon E. (2018). Moringin induces neural differentiation in the stem cell of the human periodontal ligament. Sci. Rep..

[18] Rajan T.S., Giacoppo S., Iori R., De Nicola G.R., Grassi G., Pollastro F., Bramanti P., Mazzon E. (2016). Anti-inflammatory and antioxidant effects of a combination of cannabidiol and moringin in lps-stimulated macrophages. Fitoterapia.

[19] Libro R., Scionti D., Diomede F., Marchisio M., Grassi G., Pollastro F., Piattelli A., Bramanti P., Mazzon E., Trubiani O. (2016). Cannabidiol modulates the immunophenotype and inhibits the activation of the inflammasome in human gingival mesenchymal stem cells. Front. Physiol..

[20] Rajan T.S., Giacoppo S., Diomede F., Ballerini P., Paolantonio M., Marchisio M., Piattelli A., Bramanti P., Mazzon E., Trubiani O. (2016). The secretome of periodontal ligament stem cells from MS patients protects against EAE. Sci. Rep..

[21] Cianci E., Recchiuti A., Trubiani O., Diomede F., Marchisio M., Miscia S., Colas R.A., Dalli J., Serhan C.N., Romano M. (2016). Human periodontal stem cells release specialized proresolving mediators and carry immunomodulatory and prohealing properties regulated by lipoxins. Stem Cells Transl. Med..

[22] Diomede F., Zini N., Gatta V., Fulle S., Merciaro I., D’Aurora M., La Rovere R.M.L., Traini T., Pizzicannella J., Ballerini P. (2016). Human periodontal ligament stem cells cultured onto cortico-cancellous scaffold drive bone regenerative process. Eur. Cells Mater..

[23] Rajan T.S., De Nicola G.R., Iori R., Rollin P., Bramanti P., Mazzon E. (2016). Anticancer activity of glucomoringin isothiocyanate in human malignant astrocytoma cells. Fitoterapia.

[24] Deveraux Q.L., Reed J.C. (1999). Iap family proteins—Suppressors of apoptosis. Genes Dev..

[25] Verhagen A.M., Ekert P.G., Pakusch M., Silke J., Connolly L.M., Reid G.E., Moritz R.L., Simpson R.J., Vaux D.L. (2000). Identification of diablo, a mammalian protein that promotes apoptosis by binding to and antagonizing iap proteins. Cell.

[26] Kim J.W., Choi E.J., Joe C.O. (2000). Activation of death-inducing signaling complex (disc) by pro-apoptotic c-terminal fragment of rip. Oncogene.

[27] Wolff S., Erster S., Palacios G., Moll U.M. (2008). P53’s mitochondrial translocation and momp action is independent of puma and bax and severely disrupts mitochondrial membrane integrity. Cell Res..

[28] Beurel E., Jope R.S. (2006). The paradoxical pro- and anti-apoptotic actions of gsk3 in the intrinsic and extrinsic apoptosis signaling pathways. Prog. Neurobiol..

[29] Giacoppo S., Pollastro F., Grassi G., Bramanti P., Mazzon E. (2017). Target regulation of PI3K/AKT/mTOR pathway by cannabidiol in treatment of experimental multiple sclerosis. Fitoterapia.

[30] Ozaita A., Puighermanal E., Maldonado R. (2007). Regulation of PI3K/AKT/GSK-3 pathway by cannabinoids in the brain. J. Neurochem..

[31] Brunet A., Datta S.R., Greenberg M.E. (2001). Transcription-dependent and -independent control of neuronal survival by the pi3k-akt signaling pathway. Cur. Opin. Neurobiol..

[32] Dudek H., Datta S.R., Franke T.F., Birnbaum M.J., Yao R., Cooper G.M., Segal R.A., Kaplan D.R., Greenberg M.E. (1997). Regulation of neuronal survival by the serine-threonine protein kinase AKT. Science.

[33] Kuruvilla R., Ye H., Ginty D.D. (2000). Spatially and functionally distinct roles of the pi3-k effector pathway during ngf signaling in sympathetic neurons. Neuron.

[34] Lim J.Y., Park S.I., Oh J.H., Kim S.M., Jeong C.H., Jun J.A., Lee K.S., Oh W., Lee J.K., Jeun S.S. (2008). Brain-derived neurotrophic factor stimulates the neural differentiation of human umbilical cord blood-derived mesenchymal stem cells and survival of differentiated cells through MAPK/ERK and PI3K/AKT-dependent signaling pathways. J. Neurosci. Res..

[35] Ishizuka T., Ozawa A., Katsuura M., Nomura S., Satoh Y. (2018). Effects of muscarinic acetylcholine receptor stimulation on the differentiation of mouse induced pluripotent stem cells into neural progenitor cells. Clin. Exp. Pharmacol. Physiol..

[36] Van Inzen W.G., Peppelenbosch M.P., Van Den Brand M.W., Tertoolen L.G., De Laat S.W. (1996). Neuronal differentiation of embryonic stem cells. Biochim. Biophys. Acta.

[37] Zemelko V.I., Kozhucharova I.V., Kovaleva Z.V., Domnina A.P., Pugovkina N.A., Fridlyanskaya I.I., Puzanov M.V., Anisimov S.V., Grinchuk T.M., Nikolsky N.N. (2014). Brain-derived neurotrofic factor (BDNF) secretion of human mesenchymal stem cells isolated from bone marrow, endometrium and adipose tissue. Cell Tissue Biol..

[38] Rajan T.S., Scionti D., Diomede F., Grassi G., Pollastro F., Piattelli A., Cocco L., Bramanti P., Mazzon E., Trubiani O. (2017). Gingival stromal cells as an in vitro model: Cannabidiol modulates genes linked with amyotrophic lateral sclerosis. J. Cell Biochem..

[39] Taglialatela-Scafati O., Pagani A., Scala F., De Petrocellis L., Di Marzo V., Grassi G., Appendino G. (2010). Cannabimovone, a cannabinoid with a rearranged terpenoid skeleton from hemp. Eur. J. Org. Chem..

[40] Muller C., van Loon J., Ruschioni S., De Nicola G.R., Olsen C.E., Iori R., Agerbirk N. (2015). Taste detection of the non-volatile isothiocyanate moringin results in deterrence to glucosinolate-adapted insect larvae. Phytochemistry.

[41] Diomede F., Merciaro I., Martinotti S., Cavalcanti M.F., Caputi S., Mazzon E., Trubiani O. (2016). Mir-2861 is involved in osteogenic commitment of human periodontal ligament stem cells grown onto 3D scaffold. J. Biol. Regul. Homeost. Agents.

[42] Cavalcanti M.F., Maria D.A., de Isla N., Leal-Junior E.C., Joensen J., Bjordal J.M., Lopes-Martins R.A., Diomede F., Trubiani O., Frigo L. (2015). Evaluation of the proliferative effects induced by low-level laser therapy in bone marrow stem cell culture. Photomed. Laser Surg..

[43] Diomede F., Zingariello M., Cavalcanti M., Merciaro I., Pizzicannella J., De Isla N., Caputi S., Ballerini P., Trubiani O. (2017). MyD88/ERK/NFkB pathways and pro-inflammatory cytokines release in periodontal ligament stem cells stimulated by porphyromonas gingivalis. Eur. J. Histochem..

[44] Orciani M., Trubiani O., Guarnieri S., Ferrero E., Di Primio R. (2008). Cd38 is constitutively expressed in the nucleus of human hematopoietic cells. J. Cell. Biochem..

